# Transforming Clinical Education While Addressing System Gaps: Student Leadership Experiences (SLEs)

**DOI:** 10.5334/pme.1757

**Published:** 2026-10-01

**Authors:** Amanda Binns, Farah Friesen, Ashleigh Townley, Stella Ng, Rhona Anderson, Tracy Paulenco, Kristin Collins, Lynn Sinclair, Kathryn Parker

**Affiliations:** 1 University of Toronto, Speech-Language Pathology, Toronto, ON, Canada; 2 Centre for Faculty Development, Temerty Faculty of Medicine, University of Toronto at Unity Health Toronto, Toronto, Ontario, Canada; 3 University Health Network, The Institute for Education Research, Toronto, ON, Canada; 4 University Health Network, Centre for Advancing Collaborative Healthcare and Education, Toronto, ON, Canada; 5 Holland Bloorview Kids Rehabilitation Hospital, Toronto, ON, Canada; 6 University of Toronto, Occupational Science and Occupational Therapy, Toronto, ON, Canada; 7 North York General Hospital, Toronto, ON, Canada; 8 University of Toronto, Department of Physical Therapy, Toronto, ON, Canada; 9 University of Toronto, Department of Pediatrics, Toronto, ON, Canada

## Abstract

Health and social care systems are increasingly characterized by complexity, resource constraints, and evolving service demands, requiring educational models that prepare learners to navigate and lead within dynamic environments. Student Leadership Experiences (SLEs) are proposed as a theory-informed educational model that extends beyond traditional workplace-based learning by intentionally integrating system responsiveness, collaborative leadership, and interprofessional engagement.

SLEs are structured around identified system or practice gaps and position students not only as participants in existing workflows, but as contributors to workplace-based innovation. Grounded in constructivist and transformative educational orientations, the model emphasizes dialogic engagement, adaptive expertise, and shared leadership across clinical, educational, and community partners.

This article articulates the essential elements of a Transformative SLE approach and outlines operational considerations for its enactment, including partnership development, facilitation team alignment, curriculum design, safety, evaluation, and sustainability planning. By offering a principled yet flexible framework, this work aims to support greater conceptual clarity and coherence in the design and implementation of Transformative SLEs across diverse health professions contexts.

Today’s educators prepare health professionals to work and lead in exceedingly complex and dynamic times. Student Leadership Experiences (SLEs) employ evidence-informed educational approaches to create collaborative, interprofessional workplace-based leadership development, while simultaneously addressing community needs. SLEs represent a fusion of clinical practice and education, yielding promising outcomes such as cost reduction, improved patient satisfaction, and enhanced student learning [1].

The terminology used to describe SLEs varies widely, including student-run clinics, interprofessional training wards, and service-learning activities [1,2,3,4,5,6,7]. It remains unclear whether these terms are synonymous or refer to distinct educational approaches [2]. Without clear differentiation and exploration of underlying pedagogies, essential elements, and implementation practices, educators may implement SLEs inconsistently. While all educational approaches are implemented with variation based on context and other factors (e.g., team composition, institution priorities, resource availability, learner characteristics), some degree of consistency, specificity, and well-developed rationale may inform and improve student experiences and outcomes. Research and evaluation of SLEs also requires a shared understanding across implementation contexts and instances. This article aims to outline essential elements of a particular instantiation of SLEs and considerations when designing and implementing this educational approach. As a flexible and adaptable educational model, each SLE will be unique, yet should share common principles and draw upon education theory during development and implementation.

In this article, we define the Transformative SLE approach as unique education experiences that enable collaborative learning in the context of workplace-based innovation. While traditional workplace-based learning models primarily position students within existing clinical workflows to support learning through participation, SLEs bring together health professions (HP) students and facilitation teams to develop clinical skills, build leadership capabilities, foster interprofessional team-based care, and address workplace and system gaps. Transformative SLEs position HP students as integral team members and change leaders capable of rapidly innovating in response to health and social care challenges. Students may be early in their professional development; however, they can bring novel ways of seeing to age-old problems. Developed by members of the Toronto Academic Health Science Network (TAHSN) SLE Steering Committee, [8] the Transformative SLE approach integrates evidence-informed clinical practices, education science, workplace learning, and community partnership. It aims to build clinical and leadership capacity in HP students while providing cost-effective and customised responses to system needs, such as health human resource shortages.

Transformative SLEs, like any educational innovation, should be grounded in a robust educational science evidence base. Fortunately, a wealth of experiential- and research-based evidence is available to guide their design, ensuring Transformative SLEs provide meaningful learning experiences that prepare HP students to become adaptive experts and critically reflective practitioners [9]. In developing SLEs, we draw not only from SLE-specific research [2] and experiential knowledge [10] but also from broader education theory. Two primary pedagogical approaches underpin the Transformative SLE’s development. First, we draw from cognitivist-constructivist principles, [11,12] where learning is viewed as an active process in which individuals construct and adapt mental representations by integrating new information with existing knowledge structures. These principles are used to promote the development of adaptive expertise [11,13]. Critical-constructivist principles are also foundational to this SLE approach [11,12]. These principles emphasize that knowledge construction is inherently situated within social, cultural, and historical contexts [11,12] and are used to foster critical reflection [9,14,15]. Processes aligned with this approach include critically examining power relations that shape what is known, whose knowledge is valued, and how practices are enacted [9,11,12,16]. These capabilities, that is, adaptive expertise and critically reflective practice, are essential for leading and collaborating in complex, uncertain, and value-conflicted healthcare environments, thus preparing HP students to practice and lead effectively in future health and social care challenges [13,14,15,16,17,18,19].

While SLEs are flexible and contextually responsive, several core elements distinguish them from other student-engaged or service-learning models. These essential elements include: anchoring in a clearly defined real-world system or practice gap, collaborative organisational partnership, an SLE facilitation team, education theory informed design, interprofessional and collaborative leadership structure, and embedded evaluation and sustainability planning. The sections that follow articulate the essential elements of the SLE model and then outline practical considerations for its enactment.

## Essential Elements of Transformative SLEs

Transformative SLEs are a distinct educational model defined by a set of essential elements. Together, these elements constitute the architecture of a Transformative SLE and distinguish it from traditional placements, service-learning initiatives, or student-led clinics. An educational initiative that does not include these elements may share similarities with Transformative SLEs, but would not fully reflect the approach described in this paper.

### Education Theory Informed Design

Intentional integration of education theory is a defining, foundational element of the Transformative SLE approach. Grounded in cognitivist-constructivist and critical-constructivist principles, SLEs are designed to promote adaptive expertise and critically reflective practice [9,11,12]. In contrast to models that primarily focus on the development of procedural skills, the Transformative SLE approach is intentionally oriented toward learning within complex, ambiguous, and value-conflicted environments [9,17]. Accordingly, Transformative SLE facilitators deliberately hold space for indeterminacy as a productive condition for learning, recognizing that many real-world system challenges do not have singular or pre-determined solutions.

In practice, this theoretical grounding is enacted through facilitated interactions or activities designed to foster adaptive expertise and critical reflection. For example, facilitators may introduce meaningful variation in tasks and contexts to support flexible knowledge application, [13] and create dialogic spaces that invite learners to examine assumptions, power dynamics, and decision-making processes [18,20]. See Table 1 for an example of how critical- and cognitive-constructivist education approaches are used to inform the design of a Transformative SLE.

**Table 1:** Transformative SLE Example.


ESSENTIAL ELEMENTS OF A TRANSFORMATIVE SLE	EXAMPLE OF ENACTMENT WITHIN AN AUTISM SERVICE DELIVERY TRANSFORMATIVE SLE

Education Theory Informed Design	Learning was grounded in cognitivist-constructivist and critical-constructivist principles. These principles shaped the program’s goals; namely, to foster adaptive expertise and critical reflection while supporting co-creation and collaborative leadership among health professions students. Further, it informed the approach to facilitation. Facilitation integrated clinical, educational, and lived expertise, prioritizing dialogue and collective inquiry to support co-construction of strengths-based responses to emergent needs. Educational approaches were grounded in dialogic engagement, critical reflection, and co-creation within a psychologically safe learning environment, fostering engagement with uncertainty and shared decision-making.

Anchoring the SLE in an Identified Real-World System or Practice Gap	This Student-Led Environment (SLE) initiative was developed in response to workforce capacity challenges and limited access to early identification and intervention services for autistic children and youth in Ontario. Rather than replicating existing service models, this SLE initiative adopted an innovation-oriented approach, co-created with community clinicians and families to address locally identified needs. Two complementary outputs emerged: (1) the development of a wraparound early intervention education program and (2) the creation of a tool to enhance the efficiency of early identification processes for both families and clinicians.

Facilitation Team	The facilitation team included a clinician-scientist, an education lead, and caregiver-partners, integrating clinical, educational, and lived expertise and modelling collaborative, distributed leadership.

Collaborative Organisational Partnership	The SLE was enabled through a multi-institutional partnership spanning academic, clinical, and community organizations, supporting alignment with system priorities and implementation in a real-world context.

Interprofessional and Collaborative Leadership Structure	Students from multiple health professions worked in interprofessional teams with facilitators supporting shared meaning- and decision-making. Leadership was enacted through co-creation of innovation projects aimed at addressing identified service gaps. This structure emphasized flattened hierarchies and collective responsibility for system improvement.

Embedded Evaluation and Sustainability Planning	A developmental evaluation approach was integrated from the outset to assess feasibility, acceptability, and impact within a complex and evolving service context. Feedback was gathered from learners during facilitated sessions via polls and responses to questions posed to the group. Voluntary exit interviews with individual learners were also conducted after each cohort completed the program. Evaluation findings were used iteratively to refine the Transformative SLE and inform decisions related to sustainability, including alignment with organizational priorities and potential for scale.


This table illustrates how core elements of the Transformative SLE approach are operationalized within a real-world autism service delivery context.

Rather than positioning students as passive recipients of instruction, Transformative SLEs are structured to cultivate learners who can navigate ambiguity, integrate multiple perspectives, and respond thoughtfully to evolving system demands [9,11,12]. This theoretical grounding shapes not only curriculum design but also the approach to leadership development embedded within SLEs [10].

### Anchoring in an Identified Real-World System or Practice Gap

Although workplace learning opportunities remain foundational to health professions education, [21] Transformative SLEs represent a structured extension of this approach. Traditional workplace-based learning commonly emphasizes participation within established workflows and professional socialization within existing systems (e.g., [22]). In contrast, Transformative SLEs are intentionally anchored in identified system or practice gaps and designed to position students as collaborative contributors to system improvement. This shift extends beyond student participation in real-world settings, toward structured engagement in innovation and responsive enhancement of care practices.

Building on this distinction, these gaps may relate to clinical service delivery, education, research, quality improvement, well-being, operations, etc. and are typically situated within a defined content area (e.g., stroke, autism services, post-concussion management, caregiver support). Table 1 provides an example of a real-world system gap addressed within a Transformative SLE.

### A Facilitation Team

The identified gap and content area shape the composition of the SLE facilitation team, ensuring that the expertise required to address the gap is represented. Ideally, within a Transformative SLE, an interprofessional group of students collaborate with a team of clinicians, educators, and community partners to co-design and implement innovations to the identified system need. This structure aligns with established interprofessional competency frameworks (e.g., Canadian Interprofessional Health Collaborative *CIHC Competency Framework for Advancing Collaboration* [23] and Interprofessional Education Collaborative *IPEC Core Competencies for Interprofessional Collaborative Practice* [24]). It reflects the reality that complex health and social care challenges require diverse perspectives.

At the centre of this team facilitation structure is typically an SLE Champion. Ideally, the Champion is well-versed in the gap/content area or clinical domain, familiar with workplace dynamics and system needs, and experienced in facilitation and clinical teaching. From our collective experience, Transformative SLE facilitation is enhanced by the team having knowledge and skills in interprofessional education [23,24] and collaborative leadership [23] development and education theory and practices, specifically cognitivist- and critical-constructivist principles [9]. Table 1 includes information about the Facilitation Team established for the delivery of a transformative SLE.

SLE Champions may reside within academic or clinical settings and require protected time to sustain progress and advancement of SLE projects and outcomes. In some contexts, the Champion role may be shared across individuals to enhance sustainability and distribute responsibilities. Interviews with SLE students, educators, clinicians, and community partners suggest that effective SLE Champions embody several key traits [25]:

- passion, enthusiasm, and experience with the SLE model
- insight into workplace dynamics and priorities
- ability to engage and support a broad community
- commitment to creating safe(r), brave, and accountable educational environments [26,27,28,29,30]
- commitment to supporting HP students’ learning and growth (as health and social care professionals and leaders while fostering adaptive expertise and critical reflection)

The team facilitation structure is not incidental, but rather foundational to the integrity of a Transformative SLE. Facilitation teams should reflect different sources of knowledge (e.g., clinical, research, experiential) and explicitly seek to level hierarchies that may exist within the facilitation team or between the facilitators/learners. Through explicit attention to flattening hierarchies, this role models the importance of not only including, but valuing, diverse perspectives and may provide a different experience than more didactic, “sage on the stage” educational experiences. A flatter hierarchical structure aligns with cognitivist- and critical-constructivist approaches [9,12] and establishes a different understanding of facilitator/learner relationships that may necessitate facilitators to take time to create this learning environment/relationship. It is used to create the conditions for collaborative leadership practices to be enacted, support responsible innovation within complex systems, and promote SLEs to function both as a meaningful educational experience and a mechanism for system improvement.

### Collaborative Organisational Partnership

Partnering with a community organization grounds the SLE in authentic practice realities and ensures that student learning occurs within meaningful, workplace-based contexts. Potential partners may include hospitals, community clinics, support service agencies, academic units, or other health and social care organizations (e.g. libraries, summer camps, schools).

Not all organizations will be equally suited to hosting a Transformative SLE. Given that SLEs position students as collaborative contributors and innovators, partner organizations must be receptive to shared leadership, adaptive processes, and iterative change. Understanding organizational culture, readiness for innovation, and openness to student leadership is therefore a critical early step. Furthermore, preparing partners for the expectations and structure of a Transformative SLE can enhance alignment of priorities and goals and promote sustainability. An example of organizations that came together to form a Transformative SLE is detailed in Table 1.

### Interprofessional and Collaborative Leadership Structure

Transformative SLEs are intentionally structured as interprofessional, team-based initiatives that enact collaborative leadership [23] across professional boundaries. This interprofessional orientation is foundational to how learning and leadership are organized within this model. Because the system or practice gaps addressed by Transformative SLEs are embedded within complex health and social care environments, meaningful engagement requires diverse disciplinary perspectives and shared responsibility.

Healthcare leadership is widely recognized as critical for enhancing the quality and integration of healthcare [31]. Yet, within student-engaged models such as SLEs, the nature of leadership opportunities and development approaches is often under-defined [2]. The Transformative SLE model described in this paper intentionally foregrounds ***collaborative*** leadership, aligning with the Canadian Interprofessional Health Collaborative *CIHC Competency Framework for Advancing Collaboration* [23]. This orientation aligns with critical-constructivist approaches that emphasize shared leadership, flattening hierarchies, inclusive decision-making, and relationship-centred practice.

Accordingly, students are positioned not solely as learners, but as contributors to system innovation efforts [10]. Enacting this model requires a deliberate shift in the role of preceptors and facilitators. Rather than maintaining fixed teacher-student hierarchies, facilitation teams are structurally positioned as coaches who scaffold student autonomy while ensuring appropriate supervision and accountability. This design commitment aligns with literature suggesting that autonomy can be supported through accessible supervision tailored to the learners’ developmental stage [2,32].

In this way, interprofessional collaboration and collaborative leadership operate as mutually reinforcing design commitments. Together, they shape how authority and responsibility are distributed, and how innovation is enacted within Transformative SLEs. This structural orientation distinguishes this SLE approach from traditional workplace learning models organized primarily around profession-specific participation. See Table 1 for how this was structured within an autism service delivery Transformative SLE.

### Embedded Evaluation and Sustainability Planning

Given that Transformative SLEs engage with real-world system gaps and may directly affect service users, they require mechanisms for accountability and responsible continuity. Evaluation, therefore, should be integrated from the outset of their development and implementation. Ongoing, thoughtful evaluation can play a crucial role in promoting quality clinical education and supporting sustainability efforts [33]. Together, evaluation and sustainability are essential elements of a Transformative SLE that uphold the ethical and educational integrity of the model.

Within the Transformative SLE model, evaluation serves multiple purposes: assessing educational integrity, examining system contributions, informing iterative refinement, and clarifying what constitutes meaningful success for the initiative [34,35,36]. Early articulation of the purpose of evaluation, the criteria for judging effectiveness, and the sources of credible evidence ensures alignment between educational objectives and system goals. Evaluation may draw on a range of methods, including observations, interviews, surveys, and stakeholder feedback.

Similarly, sustainability planning is embedded early in the Transformative SLE development. This is imperative when service users, such as patients and families, benefit from the SLE. Responsible design requires attention to continuity, transition, or integration of activities within organizational workflows. This may involve cultivating partnerships with like-minded organizations [37], building local capacity, embedding SLE activities within existing care pathways or curricula, or identifying funding mechanisms to support ongoing efforts. Without intentional sustainability planning, Transformative SLEs risk becoming time-limited initiatives. Refer to Table 1 for an example of an evaluation approach used within a Transformative SLE.

## From Structure to Enactment: Operationalizing the Transformative SLE Model

Translating the essential elements of a Transformative SLE into practice requires deliberate planning and coordination. While each SLE will be adapted to its local context, a series of practical phases can guide implementation. These phases are not strictly linear; rather, they often unfold iteratively as partnerships evolve and system priorities shift. The following considerations outline key steps for operationalizing the Transformative SLE model in diverse settings.

### Establish Organizational Partnership and Clarify the Gap

The Transformative SLE approach is flexible and may operate across diverse settings, including hospitals, community clinics, university-based programs, and virtual or hybrid environments. The selected partner organization and setting should align directly with the identified system or practice gap and the activities designed to address it. Considerations include organizational readiness and support, feasibility within institutional policies, accessibility for students and facilitators, and the extent to which the setting enables students to contribute meaningfully and safely.

From our experience, we have identified that Transformative SLEs often add particular value when they address areas of practice that are high-impact yet under-resourced, such as preventive care, wraparound supports, or patient and family education. Selecting a gap that is feasible, educationally meaningful for students, and responsive to organizational priorities increases the likelihood of successful implementation.

Gap identification should occur through collaborative and iterative processes that incorporate perspectives from clinicians, students, and service users. Used in the development of the autism-focused SLE (Table 1), the Design Thinking Framework [38] can guide this work. This process starts with inquiry, specifically exploring challenges within the current system, success stories, and aspirations for the future [39]. During this process, insights will be gathered from a range of voices in the system (e.g., clinicians, health service users, policy makers, students). This process aligns with recommendations within the co-production literature, which highlights processes for developing health and social care services that can facilitate cost-effectiveness, improved user and carer experience of services, and increased community capacity [40,41,42]. Example activities students could collectively lead within SLEs are provided in Table 2.

**Table 2:** Types of activities students could collectively lead within an SLE.


TYPES OF ACTIVITIES	EXAMPLES

Management/Administration	Supporting caregivers of children with complex care needs to complete forms for funding opportunities, and community support

Teaching/Education	Delivering public education about the impact of concussion at local community centres

Service/Advocacy	Delivering group social programs for patients with aphasia

Research or Evaluation Studies	Quality improvement initiatives in autism diagnosis communication across teams


Additional methods for gathering diverse perspectives may include interviews, focus groups, surveys, or informal consultation. In clarifying the gap, teams should consider community needs, learner objectives, institutional priorities, and available resources. Attention to these factors supports both contextual responsiveness and operational feasibility.

### Define Scope and Student Roles

Once the gap and partnership are established, the team should clarify the scope of student engagement and the nature of students’ contributions within the identified system context. Scope includes determining the focus, boundaries, feasibility, and anticipated level of impact of the initiative.

In parallel, intentional curriculum design translates the model’s educational commitments into structured learning experiences. This may involve sequencing tasks, embedding opportunities for structured reflection, and calibrating levels of autonomy to learners’ developmental stages. The aim is to align meaningful contribution to system priorities with the development of adaptive expertise, critical reflection, and collaborative leadership capacities. Further, intentional structuring of interprofessional interaction [23] is required. This may include clarifying role contributions, creating shared problem-solving spaces, and designing collaborative tasks that require the integration of diverse perspectives.

Inquiry into existing system processes, strengths, and constraints may inform this design process [39]. Approaches such as co-design or design thinking can support the development of contextually responsive and actionable learning experiences [38,42]. Clear articulation of expectations, responsibilities, supervision structures, and feedback mechanisms can support the coherence between educational objectives and organizational needs.

### Assemble and Align the Facilitation Team

Operationalizing a Transformative SLE requires assembling a facilitation team with complementary expertise and shared commitment to the identified system gap. Team composition may include a designated SLE Champion, clinicians, operations or administrative leads, preceptors, academic representatives, and patient or caregiver partners. Early attention to team composition ensures that both educational and organizational priorities are represented.

Once assembled, clear role delineation and shared expectations are essential. Responsibilities related to supervision, logistics, communication, and evaluation should be explicitly defined to promote coherence and accountability. Not all team members are directly involved in the day-to-day delivery of an SLE. Team members may fulfil multiple roles based on their expertise, availability, and SLE focus areas. Collectively, they contribute to the design, implementation, and sustainability. Clear role delineation early in the process enhances efficiency; however, flexibility is key, allowing roles to adapt to evolving student goals, and new initiatives or funding opportunities.

Alignment across the team can be further strengthened through intentional faculty development. Because Transformative SLEs integrate interprofessional collaboration, collaborative leadership, and theory-informed design, facilitators would benefit from preparation in these domains (e.g., CIHC, [23] IPEC [24]). Training may include strengthening collaborative practice capacities, developing skills in structured reflection and dialogue, and learning facilitation strategies that foster adaptive expertise and comfort with working within indeterminate spaces (e.g., introducing meaningful variation [13,43] and dialogue [18,20,44]. Coaching or mentorship from experienced Transformative SLE implementers can further support the facilitation team’s consistency and sustainability.

### Decide on an SLE Delivery Model and Recruit/Invite Students

Decisions regarding the SLE delivery model may influence how students are recruited and engaged. Transformative SLEs can be embedded within a core clinical placement, offered as an interprofessional education experience, or structured as an elective or additional recognition opportunity. The chosen format will shape whether participation is mandatory or voluntary, as well as how student roles and expectations are communicated.

In our experience delivering Transformative SLEs, operational considerations may include coordinating overlapping placement schedules for interprofessional student groups, clarifying processes for student assignment, and aligning evaluation and feedback requirements with institutional policies. Early attention to these logistical factors can support smoother implementation.

Recruitment processes should aim to align learner readiness with the demands of the identified system gap. Evidence suggests that matching students’ developmental stage with the opportunities and challenges of the site may enhance learning and contribution [45,46]. In considering student participation, teams may reflect on the knowledge and perspectives needed to address the gap, the learners’ level of training, program requirements, and the organization’s familiarity with the students’ professional roles.

Thoughtful alignment between student preparation, organizational context, and SLE objectives may support both educational value and system responsiveness.

### Determine Resources (e.g., Personnel, Space, Materials)

Like all education and innovation endeavours, resources are essential for developing and implementing a Transformative SLE. Importantly, these resources may be drawn from existing infrastructures, rather than requiring new investments. Space and materials can be provided in-kind or shared with partner organizations (e.g., community clinics, hospitals, academic institutions). Protected time and/or remuneration for the SLE facilitation team may be needed for designing and delivering the SLE. However, SLEs may offer a cost-effective model since team members can serve multiple purposes. For example, existing SLE preceptors often supervise more students than in a regular placement, *and* students are filling service gaps that might otherwise go unaddressed.

Even with in-kind or shared resources, funding may be crucial to establish and sustain a Transformative SLE. Consider internal and external sources, including organizational innovation and education grants, grants from health or education agencies, and government supports. However, since these grants might be time-limited, a sustainability plan is necessary. Sustainability may also be strengthened by embedding SLE activities within existing workflows, aligning initiatives with organizational priorities, and cultivating shared ownership across team members. Early consideration of how the SLE will continue beyond a single cohort can support long-term integration.

### Manage Safety and Risk

Attention to safety and risk management is an important consideration in operationalizing a Transformative SLE. Because students engage in authentic system contexts, facilitation teams should deliberately address both physical and psychological safety [47]. This may include clarifying the level of clinical supervision required for specific activities, documenting roles and procedures to support risk management (e.g., [48,49]), and providing structured orientation for both facilitators and students (e.g., [49,50,51,52]). Clear communication channels, and regular team check-ins ([50,53,54]) can further support safe implementation. Consideration of organizational policies, liability coverage [55], and scope-of-practice requirements are also advisable to ensure responsible engagement.

Of importance to note, the flattened hierarchy informed by critical-constructivist principles [12] and collaborative leadership [23] orientation of Transformative SLEs may introduce developmental tensions. Students may initially experience uncertainty or discomfort when granted increased autonomy, and preceptors may need to adjust to coaching-oriented roles. Learners accustomed to structured, instructor-led models may require additional scaffolding as they engage in process-oriented, leadership-focused activities. Creating environments in which students feel heard, supported, and able to navigate uncertainty without fear of retribution is therefore critical. Drawing from critical- and cognitive-constructivist principles, applying structured reflection, guided design approaches, and calibrated supervision within the SLE may help maintain both psychological safety and productive engagement with indeterminacy [9].

Further, to support ethical delivery of the Transformative SLE, evaluation should be integrated from the outset to support ongoing refinement and accountability [33,34,35,36,56]. Clarifying intended outcomes, identifying relevant stakeholders, and determining feasible feedback mechanisms (e.g., structured reflection, stakeholder surveys, observational data) can enable iterative adjustment.

## Conclusion

Transformative SLEs represent a proposed educational approach that extends beyond traditional workplace learning by intentionally integrating system responsiveness, collaborative leadership, and theory-informed curriculum design. Rather than positioning students solely as participants within existing structures, SLEs are designed to engage learners as contributors to addressing real-world gaps within health and social care systems. In doing so, SLEs emerge as a promising education model with the potential to transform clinical training and practice.

In this paper, we have outlined essential elements and operational considerations that may guide educators and organizations seeking to enact this model. Grounded in educational theory and informed by collaborative, interprofessional approaches (e.g., [54]), Transformative SLEs aim to cultivate adaptive expertise, critical reflection, and collaborative leadership capacities while responding to identified community and organizational needs.

As health systems continue to navigate complexity, resource constraints, and evolving care demands, education approaches that intentionally integrate learning with system improvement may hold promise. Further empirical studies are needed to examine the impact, feasibility, and sustainability of Transformative SLEs across contexts. We offer this framework as a starting point for dialogue, refinement, and scholarly exploration.
